# Infants who are rarely spoken to nevertheless understand many words

**DOI:** 10.1073/pnas.2311425121

**Published:** 2024-05-30

**Authors:** Ruthe Foushee, Mahesh Srinivasan

**Affiliations:** ^a^Department of Psychology, New School for Social Research, New York, NY 10011; ^b^Department of Psychology, University of California, Berkeley, CA 94705

**Keywords:** child-directed language, overhearing, word-learning, cross-cultural, language socialization

## Abstract

Theories of language development—informed largely by studies of Western, middleclass infants—have highlighted the language that caregivers direct to children as a key driver of language learning. However, some have argued that language development unfolds similarly across environmental contexts, including those in which childdirected language is scarce. This raises the possibility that children are able to learn from other sources of language in their environments, particularly the language directed to others in their environment. We explore this hypothesis with infants in an indigenous Tseltal-speaking community in Southern Mexico who are rarely spoken to, yet have the opportunity to overhear a great deal of other-directed language by virtue of being carried on their mothers’ backs. Adapting a previously established gaze-tracking method for detecting early word knowledge to our field setting, we find that Tseltal infants exhibit implicit knowledge of common nouns (Exp. 1), analogous to their US peers who are frequently spoken to. Moreover, they exhibit comprehension of Tseltal honorific terms that are exclusively used to greet adults in the community (Exp. 2), representing language that could only have been learned through overhearing. In so doing, Tseltal infants demonstrate an ability to discriminate words with similar meanings and perceptually similar referents at an earlier age than has been shown among Western children. Together, these results suggest that for some infants, learning from overhearing may be an important path toward developing language.

## Significance

Decades of research—largely conducted in Western, child-centered contexts—have highlighted the speech that parents direct to children as the primary driver of language development, as opposed to the speech that children overhear. This conclusion has driven interventions globally to encourage parents to speak more to their children. Yet there is dramatic cross-cultural variation in how much adults speak to infants, raising the question of how children robustly learn language across contexts. We show that Tseltal infants—who are rarely spoken to—show knowledge of common nouns, as well as knowledge of greeting terms they could *only* learn through overhearing. These findings suggest that, for some infants, learning from overhearing may be critical to developing language.

One of the most remarkable aspects of human development is that, across widely different environments, cultural settings, and approaches toward child rearing, children rapidly acquire their native languages (1–3). Yet although language acquisition is admired for its robustness across diverse contexts, theories of language development—and of how the environment might facilitate learning—have been informed largely by studies of children raised in Western, middle-class, child-centered households (3–6). In studying the efficacy of features of the early language environment in primarily Western settings, researchers have suggested that there may be a single, optimal pathway to language (7; or indeed, any sort of cultural learning: 8–10)—one that involves frequent adult speech to children, even before children can meaningfully reply (*child-directed language*). Decades of research in this tradition have highlighted language occurring within one-on-one interactions between caregivers and young children as the primary driver of language development (11–14), suggesting that increases in the quantity and quality of child-directed language lead to increased language processing efficiency, vocabulary growth, optimal brain development—and ultimately, increased educational attainment—for children (15–23). This body of work has led some to characterize sparsity of child-directed language as a form of early deprivation (24) and has spurred interventions globally to encourage caregivers to speak more to their young children (25).

At the same time, a parallel intellectual history in anthropology challenges the assumption that language development critically relies on child-directed language, by documenting that, in spite of wide global variation in child-directed language customs (26–40), children appear to develop language on similar timetables across contexts (2, 41–44). These correspondences in the emergence of language are remarkable, given that variation in child-directed language can be quite stark across communities: one study, for example, found that toddlers in Chicago received 11 times more child-directed utterances (including both speech from adults and other children) than did their peers from a Yucatec Mayan village (31).^*^ Yet even within contexts in which child-directed language is sparse, children are often surrounded by ample speech—directed to others in their environment—that they could, in principle, *overhear* (45). In the Yucatec Mayan community referenced above, up to 80% of the utterances in toddlers’ environments were directed to others (31). And another study found that, for children from Rossell Island in Papua New Guinea, 91% of speech was other-directed (46).

These observations, taken together with reports that children across diverse language environments reach basic language milestones at similar ages (41), raise the question of whether children who receive little child-directed language are able to tune into and learn from speech directed to others around them (34, 35, 37, 42, 45, 47–50). The present studies address this question by probing early knowledge of words—including honorific greeting terms that are never addressed to children—among infants from an indigenous community in Southern Mexico who are rarely spoken to directly.

Competing claims regarding the roles of child-directed and other-directed language in language development have been difficult to evaluate because they rely on evidence derived from distinct disciplines and methodologies. Evidence that language acquisition depends critically on child-directed language has largely come from quantitative studies in developmental psychology, which have typically measured lexical knowledge, or vocabulary, as a correlate of variation in children’s environments (13, 18, 20, 44, 51; see ref. 52 for a review). On the other hand, evidence that children who receive little child-directed language develop language on a similar timetable to children who are more frequently spoken to has derived primarily from descriptive work in linguistic anthropology (2, 35, 41, 42); thus, there has been little *quantitative* evidence to address whether children who receive sparse child-directed language develop linguistic knowledge in part by learning from the speech that they overhear in their environments—and the work that has been done has not yielded clear conclusions (31, 43). Gathering data to bear on this hypothesis—a primary goal of our studies—is critical to understanding what counts as effective input for developing language across diverse developmental contexts.

Although a significant body of experimental work suggests that by age two, children can learn the meanings of new words by observing third-party interactions (53; see also ref. 54), it is unclear how these experimental demonstrations bear on younger children’s ability to learn from other-directed speech “in the wild” (11, 55), which we focus on here. In most of these studies, children only needed to learn a single word, which was typically repeated many times and stressed, embedded in explicit labeling frames, presented using the prosody and intonation of child-directed speech, and supplemented with overt cues to reference like eye gaze and pointing (for a review, see ref. 55). Thus, the other-directed speech presented in these studies is unlikely to be representative of the other-directed language in children’s real-world environments, which will often lack these features. More generally, learning from other-directed language in the real world will often require children to inhibit their own focus of attention to instead attend to and make sense of an interaction among others; yet in prior experiments, these demands were minimized such that there was little beyond the third-party interaction competing for children’s attention (11). Perhaps consistent with these concerns regarding the external validity of prior overhearing experiments, no studies have found that the quantity of speech that children could overhear in their real-world environment predicts their subsequent vocabulary growth, despite finding robust analogous correlations between the quantity of child-directed language and vocabulary growth (31, 44).^†^

**Fig. 1.**
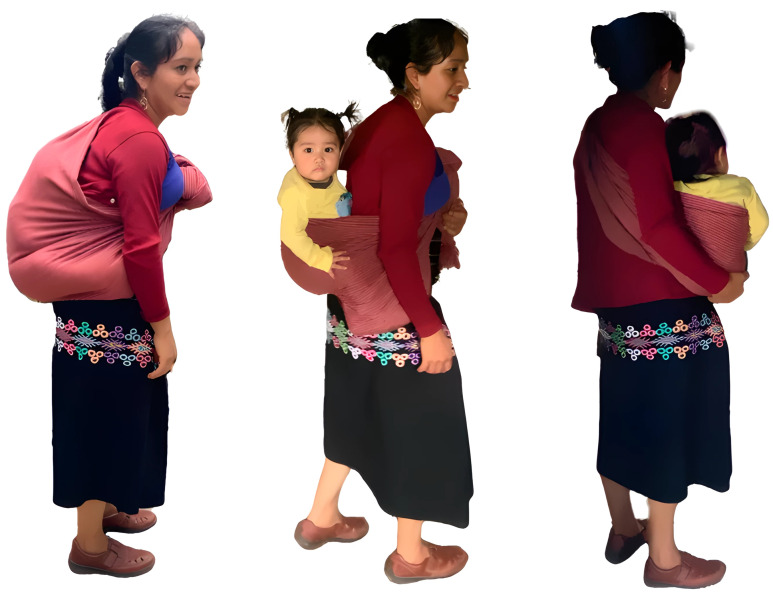
Tseltal mother carrying nine-mo-old infant.

Based on these and other considerations, there has been a general consensus within developmental psychology that child-directed language is the primary driver of language learning, with other-directed language playing a marginal role, if any role (11, 60). Indeed, Golinkoff and colleagues write that “...there are no data to suggest that overheard speech promotes language learning during the period when children are first breaking into language” (11, p. 987). The present studies address the hypothesized universal role of child-directed language in lexical development by testing whether a group of infants who are rarely addressed directly nevertheless exhibit early lexical knowledge, including knowledge of language that could *only* have been learned through overhearing. We employ measures that are specific to the developmental context of a Tseltal Maya society in Tenejapa, Chiapas, Mexico, where infants experience the world from a sling on their mothers’ backs (Fig. 1).

Importantly, our goal was *not* to construct a dataset directly comparing Tseltal-learning infants with, e.g., English-learning infants. Any apparent differences in the learning trajectories of Tseltal infants relative to infants in other communities could be due to countless factors, only one of which is the nature of their language environments. Instead, our study was inspired by observing that a specific theory has emerged regarding the environmental conditions that best support (all) children’s learning of new words—i.e., that words are primarily learned through accumulating experience with those words in child-directed language (11, 25, 60–62)—and that this theory predicts that Tseltal infants should have minimal lexical knowledge, given that they are rarely the direct recipients of speech. Evidence that infants in this context and developmental period possess such lexical knowledge would thus be a challenge to prevailing theory.

## Context of the Present Studies.

Several details of Tseltal infants’ socialization context motivate the present study as an ideal test of children’s ability to learn from the other-directed language in their environments.

First, anthropologists have noted that Tseltal infants are very rarely directly addressed by caregivers for the first year or so of life, in part because they are almost always carried on their mothers’ backs (36, 63). Multiple ethnographies indicate that it is only in later infancy—after they begin walking, interacting with a broader set of caregivers, and speaking themselves (35, 41)—that Tseltal infants more frequently become the direct recipients of speech. In support of the claim that Tseltal children receive little directed speech—and much less speech than their North American peers—one study estimated that Tseltal children under age 3 were spoken to by adults or other children for an average of 3.63 min/h (compared to hearing 21 min of other-directed speech/h; 43), which is at least three times less than analogous estimates from communities in the U.S. and Canada (64). Similarly, another study found that while toddlers from a Yucatec Mayan community heard 55 utterances/h directed to them by adults or other children, toddlers in Chicago heard 605 child-directed utterances/hour, an 11-fold difference (31).

Ethnographic work (34) further suggests that, when Tseltal caregivers *do* speak to their infants, it may be primarily to control their behavior and movements (e.g., via brief imperatives like “don’t cry” or “go to sleep”). Tseltal caregivers are unlikely to interact with infants pedagogically (e.g., labeling the focus of infants’ attention or asking known-answer questions) or view them as conversational partners (e.g., by responding contingently to infants’ babbling)—practices that have been associated with growth in children’s language skills within Western contexts. Together, these considerations suggest that if Tseltal infants rely solely on the child-directed input in their environments to learn—as suggested by current theories—they should have minimal lexical knowledge due to the limited quantity and particular character of this input^‡^; conversely, if Tseltal infants do exhibit significant lexical knowledge, this knowledge is likely to have been derived at least in part from “listening in” to other-directed language.

Second, Tseltal infants in slings on their mothers’ backs find themselves in an ideal position to learn from other-directed language, having a “front-row seat” to their mothers’ interactions (Fig. 1). While these infants are rarely spoken to, they are also almost never put down, or even passed to siblings, ensuring that they are witness to practically the entirety of their mothers’ social interactions. Certainly when peeking over her shoulder, and possibly even when wholly enshrouded in the sling on her back, infants will receive high-quality audio, and have at least some access to the focus of their mothers’ attention, which could suggest the referents of her and others’ speech.

Third and finally, infants in this community are exposed to several distinctive social speech signals that are never directed to them, yet exhibit many of the features thought to make infant-directed utterances conducive to learning. Specifically, greetings among adult community members are frequent (72), highly ritualized (73, 74), and delivered at high volume, with a unique prosodic contour (23, 75) over the relevant term. The specific honorific terms used in these greeting exchanges depend on the sex, relative age, and sometimes familial relation or authority of the speakers (*SI Appendix*, Text). Importantly, as there is no way to greet an *infant* using this system, these honorifics represent language that could *only* be learned by overhearing.

## Methodological Approach.

Prior studies aiming to assess the lexical knowledge of learners who receive little child-directed language have typically relied on parental vocabulary reports or verbal tests of children’s expressive and receptive vocabularies. However, these explicit tasks—in which information about the child’s linguistic knowledge relies on explicit verbal and/or behavioral responses from the caregiver or child—may be less sensitive in contexts where one-on-one verbal interactions between adults and children are infrequent. From this perspective, measuring vocabulary comprehension by asking a child to point to the correct picture for a given word (31) may feel unnatural to children who are infrequently engaged directly (7, 35, 37, 41, 48). Likewise, asking a caregiver to complete a checklist of their child’s expressive vocabulary or linguistic milestones (76–78) might systematically underestimate the language knowledge of children who are not encouraged to speak, as their caregivers will have fewer opportunities to realize the extent of their knowledge.

In light of these concerns, we used an *implicit*, gaze-based measure of language knowledge (80, 81). Specifically, we adapt and extend a well-established “language-guided-looking” task, which captures early word knowledge by measuring infants’ gaze between paired visual stimuli when prompted with one of their names (81–86). On each trial, infants were presented with two discrete images, one of which was labeled by their caregivers, who heard the relevant prompt through headphones and repeated it aloud. Importantly, the format of the task ‘spared’ infants from unfamiliar face-to-face interaction, making it more compatible with the indirect ways in which Tseltal infants are accustomed to interacting with adults (36, 37).

**Fig. 2.**
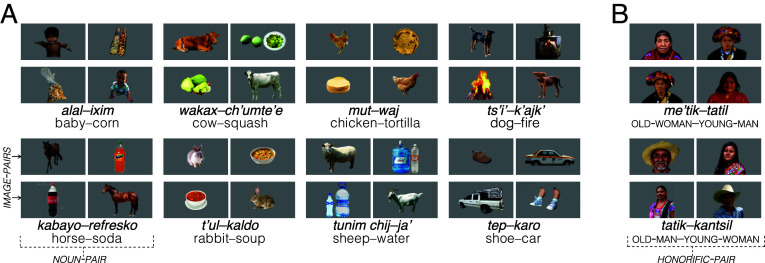
Paired-picture stimuli used in Experiments 1 and 2. Infants saw two image-pairs for each noun-pair in Experiment 1 (*A*) or each honorific-pair in Experiment 2 (*B*).

Prior research employing this method has found that U.S. infants exhibit knowledge of common English nouns (82, 87). Experiment 1 thus tested whether Tseltal infants exhibit analogous knowledge of common Tseltal nouns (mostly names for food, objects, and animals; see Fig. 2). We reasoned that if current theories are correct, and all children rely primarily on child-directed language (as opposed to other-directed language) to learn new words, Tseltal children’s word learning should be significantly delayed relative to U.S. infants—by virtue of being spoken to far less often—to the extent that they should fail to show evidence of analogous lexical knowledge in infancy. This prediction follows from much prior work, which has documented differences in vocabulary knowledge across groups and attributed those differences to disparities in experience with child-directed language (44, 88, 89). For example, one study found that by 24 mo of age, a sample of low-SES U.S. English-speaking children were 6 mo behind their higher-SES peers (using a similar measure to the one used here), a disparity that the authors attributed to differences in the quantities of speech directed to these children (90).^§^

Experiment 2 then builds on Experiment 1 to more directly test learning from overheard speech. We probed infants’ knowledge of language that they could only learn by overhearing—i.e., the core set of honorific greeting terms used in Tseltal.

## Results

### Experiment 1: Common Noun Knowledge.

Following previous work (82), we operationalize infants’ word recognition, based on their gaze data, in two ways. First, we compute a pair-based measure: For each noun-pair (e.g., *baby-corn*), we take the proportion of an infant’s looking time that was directed to a particular image (e.g., the baby) on the trial when that image was the target (e.g., when the prompt was to look at the baby), and subtract from it the infant’s proportion looking time to that same image on a matched trial when it was *not* the target (i.e., when the prompt was to look at the corn); the infant’s difference score for this noun-pair would be the mean of this value computed for both *baby-corn* image-pairs. The benefit of this metric lies in its interpretability—positive values suggest word knowledge—and its correction for possible baseline visual preferences for one image over another. But this benefit also comes with a cost, as difference scores can only be calculated for image-pairs in which the infant provides usable data on *both* presentations of the pair. Thus, our second, complementary analysis models infants’ *within*-trial looking time patterns—specifically capturing whether infants’ preference for the target image reliably increases upon hearing the target word during each trial. This approach enables us to use data from all nonexcluded trials for every child.

#### Pair-based mean difference scores.

All (8/8) noun-pairs showed positive mean scores, suggesting that the infants in our sample had knowledge of the nouns we tested, despite receiving very little directed speech (range =0.03to0.23, M=0.12, 95% bootstrapped CI=[0.07, 0.17]; P<0.01, Wilcoxon test; P<0.01, binomial test; Cohen’s d=1.57^¶^; *SI Appendix*, Table S1 andFig. 3*A*). The mean of infants’ subject-level scores was also positive (range =−0.20to0.45, M=0.11, 95% CI=[0.04, 0.18]; P<0.01, Wilcoxon test; P<0.01, binomial test; Cohen’s d=0.64), with a majority (17/21) of infants (*M*_age_ = 11.19 mo, SD_age_ = 2.74 mo) showing positive means across noun-pairs (*SI Appendix*, Fig. S5*A*). A linear mixed effects model with random intercepts for subjects (92) indicated that these results were not driven by a few exceptional infants ($\beta_{0}=0.12$, 95% CI=[0.04, 0.20]; t(22.91)=3.10, P<0.01; $\chi^{2}\left(1\right)=7.89$, P<0.01; Cohen’s d=0.27; *SI Appendix*, Table S3).

**Fig. 3.**
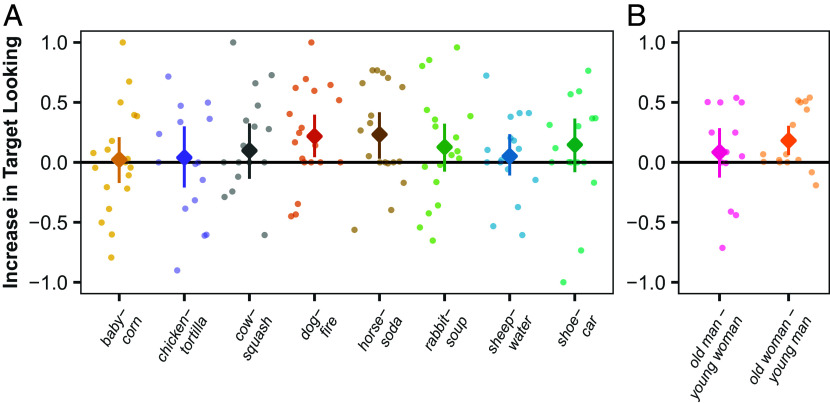
Item means in Experiments 1 and 2. Mean pair-based difference scores in paired-picture trials testing infants’ knowledge of high-frequency Tseltal nouns in Experiment 1 (*A*) and honorifics in Experiment 2 (*B*). Transparent dots plot individual participant data; solid diamonds plot means across participants (see *SI Appendix*, Tables S1 and S2 for values). Vertical lines indicate 95% bootstrapped CI.

Subject-level scores exhibited an insignificant positive correlation with infants’ age in months (τ=0.25, P=0.116).^#^

The evidence that Tseltal infants possessed knowledge of the words we probed is similarly strong to evidence that has been used to infer the presence of word knowledge among U.S. infants of different ages (*SI Appendix*, Fig. S5). For example, Bergelson and Swingley (82) reported mean scores of 0.074 (over subjects) and 0.065 (over items) for 6- to 9-mo-olds, 0.055 (over subjects) and 0.059 (over items) for 10- to 13-mo-olds, and 0.29 (over subjects) and 0.28 (over items) for 14- to 16–mo-olds. We found mean scores of 0.04 (over subjects) and 0.03 (over items) for 5- to 9-mo-olds, 0.13 (over subjects) and 0.12 (over items) for 10- to 13-mo-olds, and 0.25 (over subjects) and 0.26 (over items) for 14- to 16-mo olds.

#### Looking-time preference by trial phase.

If infants knew the words we tested, we expected they would dedicate a greater proportion of their looking time to the target image after hearing the target word. We tested this prediction via a mixed effects logit model fit to the trial-by-trial ratio of infants’ looking times (in number of 20 ms bins; see ref. 82) to the target vs. the nontarget image, with trial phase (PRE-/POSTNAMING; see Fig. 5) as a fixed effect, and random intercepts for both subject and item (*SI Appendix*, Text).^‖^

The odds ratio for trial phase (POSTNAMING OR=1.28, 95% CI=[1.26, 1.31]) indicates that infants dedicated a significantly greater share of their visual attention to the target image *after* hearing it labeled than *before* hearing it labeled, controlling for subject- and item-level variability (Wald $\chi^{2}\left(1\right)=641.07$, P<0.001; Cohen’s d=1.05; see *SI Appendix*, Table S5 andFig. 4). A model which additionally included infant age and its interaction with trial phase resulted in a significantly better fit ($\chi^{2}\left(2\right)=401.95$, P<0.001), showing a reliable effect of trial phase (OR=1.29, 95% CI=[1.26,1.31]; Wald $\chi^{2}\left(1\right)=635.84$, P<0.001; Cohen’s d=1.07) and interaction between trial phase and age, such that older children showed a greater increase in the ratio of target to nontarget looking after hearing the target word (OR=1.08, 95% CI=[1.07, 1.09]; Wald $\chi^{2}\left(1\right)=401.02$, P<0.001; Cohen’s d=0.32). This effect of age suggests that infants’ word recognition performance benefited from having heard more (other-directed or child-directed) speech and/or from increased cognitive maturity.

**Fig. 4.**
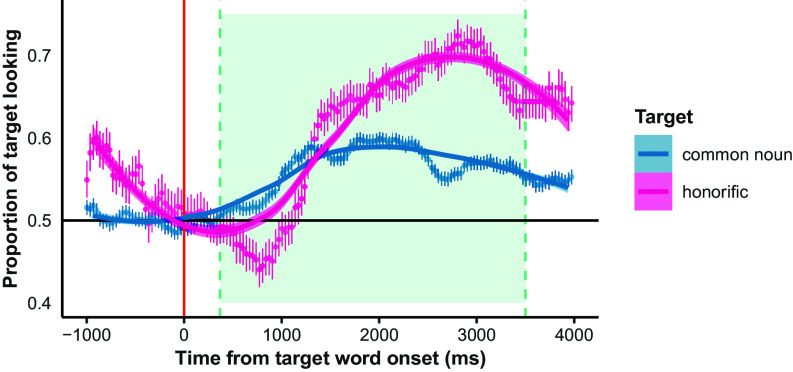
Timecourse of infant looking in Experiments 1 and 2. Points plot the proportion of infants looking to the target image (out of the total infants looking to either image) at each 33 ms time interval, with error bars indicating 95% bootstrapped CI, for Experiment 1 (common nouns; blue) and Experiment 2 (honorifics; fuchsia). The vertical line (in red) marks the onset of the target word, and the shaded region indicates the analysis window, from 367 to 3,500 ms post-target-onset. The overlaid line represents a loess model (79) fit to the binned proportion data, weighted by the number of infants in each bin.

Together, the results of Experiment 1 suggest that our sample of Tseltal infants—who are rarely spoken to directly—nevertheless understood many common nouns. It is, of course *possible* that they developed this knowledge exclusively from the small amounts of child-directed language they have received. Yet, as reviewed in the introduction, prior findings and current theory strongly predict that disparities in the quantity of high-quality speech directed to children will be a key determinant of differences in the levels of vocabulary knowledge that children develop (90). From this perspective, Tseltal infants should have been unlikely to exhibit analogous lexical knowledge to their U.S. peers, who are spoken to much more frequently. At minimum, our results suggest that if current models are correct—and all children rely primarily on child-directed language to learn—then the quantity of child-directed language that infants receive does not make a substantial difference to the scale of lexical knowledge that they develop. This would represent a major departure not only from prevailing theory, but also from widespread interventions founded on this theory, which encourage parents to speak more to their young infants.

Instead, we suggest that the lexical knowledge demonstrated by Tseltal infants in Experiment 1 was learned in no small part from overhearing. However, this inference is necessarily indirect. Experiment 2 provides a more stringent test of learning through overhearing, by asking whether Tseltal infants show knowledge of words that they could *only* have encountered in language directed to others.

### Experiment 2: Honorific Knowledge.

As previously noted, honorific greeting terms in Tseltal are never directed toward infants. Thus, infants’ positive recognition of these terms would necessarily reflect knowledge acquired via overhearing.

**Fig. 5.**
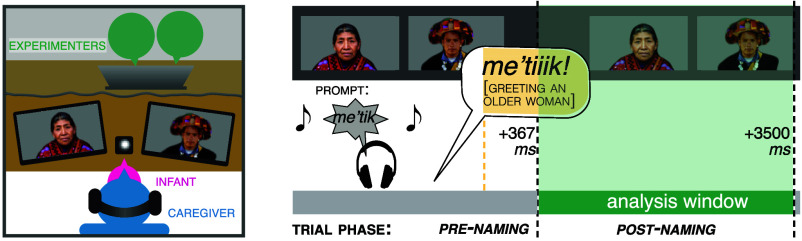
Experimental setup and trial structure for Experiment 2. Experiments take place in a felt tent. Experimenters control stimuli from behind a curtain. Infants sit on caregivers’ laps; caregivers repeat prerecorded prompts, while infants’ gaze is recorded.

We tested honorific knowledge using the same method as in Experiment 1: Mothers were prompted to repeat an honorific greeting term appropriate for addressing one of the two faces that infants were presented on each trial^**^ (Fig. 5).

Experiment 2 invites two competing predictions regarding infants’ performance: on the one hand, we might expect high performance, because honorific greeting routines are frequent and perceptually salient, and because their real-world call-and-response format might naturally trigger infants’ gaze to the target face (i.e., in anticipation of the addressee’s response). On the other hand, the extant literature strongly predicts that infants should *not* demonstrate knowledge of honorifics in this task until significantly later in development (86, 87, 93, 94). Prior work with English-learning U.S. infants finds that infants younger than 18 moshow no gaze preference for an image (e.g., of a cookie) that precisely matches a target word (e.g., cookie), relative to an image that is merely related to it (e.g., of a banana; 87, 93), or whose label has a similar word frequency (86). And even older English-learning infants (18 to 24 mo) fail to disambiguate two familiar entities if they are both semantically related *and* perceptually similar (e.g., *apple–orange*; 94). These findings predict that Tseltal infants should be highly unlikely to show honorific knowledge in Experiment 2, since the task pairs perceptually similar images (faces) from the same semantic category (HUMAN), and prompts infants’ attention between them using semantically related words (honorifics: *me’tik, tatik, kantsil, tatil*), which have highly similar frequencies and contexts of use.

#### Pair-based mean difference scores.

Both honorific-pairs showed positive mean difference scores (range =0.08to0.18, $M_{=}0.13$, 95% CI=[0.08, 0.18]; P<0.05, Wilcoxon test; P=0.50, binomial test; *Cohen’s* d=1.95; Fig. 3*B*), suggesting that infants overcame the perceptual similarity and semantic relatedness of the test stimuli to look to the correct addressee for each honorific. The mean across subjects was also positive (range =−0.36to0.54, M=0.14 95% CI=[0.01, 0.28]; P=0.095, Wilcoxon test; P=0.118, binomial test; Cohen d=0.52; *SI Appendix*, Fig. S5*B*), though not significant, with 11/15^††^ subjects showing positive difference scores (*M*_age_ = 10.88 mo [9.16, 12.43], SD_age_ = 3.09). The intercept of a linear mixed effects model accounting for subject-level variability suggested that infants’ mean performance was positive, but this was not significant ($\beta_{0}=0.14$, *95% CI*=[−0.01,0.29]; t(15.23)=1.98, P=0.066; $\chi^{2}\left(1\right)=3.53$, P=0.060; *Cohen’s*d=0.43; *SI Appendix*, Table S4). Mean scores were also not significantly correlated with infants’ ages in months (τ=0.05, P=0.85).

We note that the analyses in this section only permit inclusion of data from trials where an infant contributed usable data for both presentations of a given image-pair. The logit models presented below enabled inclusion of n=25 additional trials (22% of all nonexcluded trials), including data from three additional children, thereby allowing for a more comprehensive evaluation of the evidence.

#### Looking-time preference by trial phase.

We fit a mixed effects logit model to children’s ratios of pre- versus postnaming looking times (in terms of number of 20 ms bins), with random intercepts by subject (N=21). The odds ratio for trial phase (POSTNAMING OR =1.30, 95% CI=[1.24,1.36]) suggests that infants’ relative looking time to the competitor faces was responsive to the honorific term used by their caregivers: Infants dedicated a significantly greater proportion of their looking time to the target face after hearing the honorific (Wald $\chi^{2}\left(1\right)=123.99$, P<0.001; Cohen’s d=0.56; *SI Appendix*, Table S6, Text andFig. 4). As in Experiment 1, a model which additionally included infant age and its interaction with trial phase resulted in a significantly better fit ($\chi^{2}\left(2\right)=18.32$, P<0.001; *SI Appendix*, Table S6), showing a reliable interaction, such that older children showed a greater increase in the ratio of target to nontarget looking after hearing the target word (POSTNAMING: Age OR=1.03, 95% CI=[1.02, 1.05]; Wald $\chi^{2}\left(1\right)=13.82$, P<0.001); however, the size of the effect was very small (*Cohen* d=0.08). The weaker age effect relative to Experiment 1 could reflect the high frequency of honorifics in daily life (such that younger infants may have encountered them sufficiently often), and/or the increased ecological validity of the method of Experiment 2 relative to Experiment 1 (which could have lowered task demands to the point that even younger infants could succeed).

## General Discussion

Decades of research on language development have coalesced around the idea that infants around the world are able to break into language and begin learning words in large part due to the rich, simplified speech that their caregivers direct to them (11–14, 17, 18, 20, 44, 52). This consensus predicts that infants who are surrounded by ample other-directed language but who are rarely spoken to early in life—like Tseltal Maya infants—should exhibit minimal lexical knowledge. Adapting an implicit, gaze-tracking measure to our field setting, we conducted two studies which provide evidence against this prediction. Experiment 1 deployed a task used to show early knowledge of common English nouns among U.S. infants, and showed that Tseltal infants exhibit analogous knowledge of common Tseltal nouns, despite receiving far less child-directed language than their U.S. peers. Supporting the idea that Tseltal infants derive their early lexical knowledge in part from other-directed language, infants in Experiment 2 showed knowledge of Tseltal honorific greeting terms that are exclusively used between adult members of the community and could thus only be learned via overhearing.

Together, these findings provide evidence that infants can and do learn from the other-directed language in their environments, and suggest that learning via overhearing may be an important path toward developing language for some infants, contrary to prior claims that other-directed language is of little value to young language learners (11). Language development is often admired for its robustness and resilience across variable environmental contexts (1, 3), including contexts in which infants are rarely addressed directly. Our findings invite the perspective that children successfully acquire their native languages across variable environments in part because they are flexible in how they learn—i.e., they are able to learn not only from the language directed to them but also from the language that they overhear.

A key question opened by our findings is whether, across different environmental contexts, children are equally attentive to and able to learn from child-directed and other-directed language, or whether children might instead develop and adapt their learning strategies in response to their environments. By the latter account, children raised in child-centered contexts may come to expect their attention to be managed by their caregivers and learn to ignore interactions around them^‡‡^; conversely, children raised in contexts in which child-directed language is rare and other-directed language is common may develop a keen ability to attend to and learn from interactions around them (34, 35, 37, 41, 48–50). To directly test the adaptation hypothesis, future studies could measure the composition of child-directed and other-directed language in children’s environments and explore how this relates to children’s attention to and propensity to learn from these different informational sources, as well as how this relation might emerge over development (i.e., as children gradually “learn to learn” from their environments). Future work could also benefit from further attention to the different kinds of other-directed language that children experience (e.g., speech to another adult vs. another child), and whether some kinds of other-directed language are more conducive to learning than others (cf. refs. 59 and 96).^§§^

Our study joins recent and ongoing efforts to characterize the scope of diversity in language environments and potential language learning mechanisms (33, 43, 45, 59, 97–101). Because most prior work has been conducted in contexts where child-directed language is common, it is perhaps unsurprising that this work has focused on how and what children learn from child-directed language, and has highlighted such language as critical to learning. Our studies show how considering how learning proceeds within other contexts of development can shift our views about the informational sources and learning mechanisms that children employ to learn language. For example, although young children are sometimes cast as the passive recipients of adult language input and guidance, Tseltal infants’ apparent ability to listen into and learn from other-directed speech arguably constitutes a strength (102), and highlights the active role that children may play in their own language development (103). Our findings may thus be relevant for interventions which encourage caregivers to speak more to their young infants—particularly in contexts in which this practice is not the cultural norm (25).

To conclude, our research with infants growing up in Tenejapa, Chiapas was designed to shed light on the tension between ethnographic descriptions of child development across the world—which emphasize that all children come to speak the language of their communities—and quantitative studies from developmental psychology, which have reached conclusions about the primacy of child-directed language based largely on data from children from Western, child-centric households. In showing that infants who are rarely spoken to can develop early lexical knowledge—including knowledge that could *only* be learned through overhearing—our work illustrates how flexibility in how children learn may help explain the robustness of language acquisition across variable human environments.

## Materials and Methods

### Experiment 1.

Experiment 1 used a gaze-based metric of word recognition to measure infants’ knowledge of common Tseltal nouns.

#### Participants.

We report on data from 21 infants (5.33 to 15.43 mo, *M*_age_ = 10.96 mo, SD_age_ = 2.71 mo) tested in the Cañada Chica *paraje* of a Tseltal Maya community in Tenejapa, Chiapas, in Southern Mexico. Mothers (14–44 y, *M*_age_ = 26.85 y, SD_age_ = 8.36 y) spoke Tseltal as their primary language, and reported carrying their infants on their backs for the majority of the day, removing them only to sleep and to bathe. Critically, the infants whose data we analyze here had not yet begun to walk—a milestone that previous interviews and observations suggest marks the onset of a significant increase in directed speech, and that coincides with increases in child-directed speech reported in similar contexts (31; though see ref. 43). Participants were recruited by a research assistant from the community and the first author, who called at family compounds within a 2-h hike of the testing location to ask after new mothers (*SI Appendix*, Text).

Dyads often participated in multiple experiments during their data collection visit: Experiment 1 was always infants’ first task, followed by Experiment 2, and/or pilots of other tasks in development. In addition to experimental data, we collected daylong audio recordings for 18 infants in our sample, enabling future analyses of their naturalistic language environments at the time of the study.

Caregivers received 50 MXN per task as compensation, approximately the rate for half of a day of field labor. This amount is appropriate given the significant time investment required for mothers to walk to and from the study site—often with multiple children—and given that participation removed them from other necessary or lucrative activities. All protocols were approved by the University of California, Berkeley Committee on the Protection of Human Subjects. We conducted informational interviews with mothers of young infants and took photographs for experimental stimuli in March 2019, and collected data in January 2020.

#### Stimuli.

Visual and auditory stimuli tested infants’ knowledge of eight noun-pairs composed of high-frequency, imageable nouns. Nouns referred to entities that were frequent and/or salient in infants’ environments—including domesticated animals and foods associated with babies—and were reported as among the first words children produced. Paired nouns typically differed in animacy (Fig. 2).

Many of the visual stimuli were created using photographs taken within the community; the remaining photos were selected from among publicly available images, in consultation with local research assistants. To ensure that the photos we chose were good exemplars of the nouns they were meant to depict, we recruited a sample of adults (N=9) from the community to participate in a picture-naming task. We used only photos that all adults spontaneously labeled with the intended noun (*SI Appendix*, Text for more detail). These were then edited using Adobe Photoshop (20.0.0) to create 32 standardized visual stimuli featuring a target image centered on a 16 × 9 monochrome gray background (Fig. 2). Paired images were adjusted to approximately equate their brightness and area. There were two image-pairs for each noun-pair, with each image serving as the target on one trial (32 total trials: 8 noun-pairs × 2 image-pairs × 2 targets; see *SI Appendix*, Fig. S1).

The audio stimuli consisted of prompts embedding each noun in one of four carrier sentences, e.g.: *banti ay te ch’uhmate’e?* (“where is the squash?”); *ilawil te tunim chij* (“look at the sheep”); *inba’ay kojt karo* (“there is a car”); *yabal stak’ tajtik te me t’ule?* (“can you find the rabbit?”). Prompts were recorded by an adult native speaker speaking at a rate of 3 to 4 syllables/s, and later standardized for volume and length (2 s). Over the course of the experiment, each noun-pair was prompted using two carrier sentences. Trials were presented in a pseudorandom order designed to maximize distance between repeated carrier sentences and noun-pairs.

#### Apparatus and procedure.

Caregivers verbally consented to participate, then shared their privacy concerns in a semistructured interview. Next, they were familiarized with all experimental stimuli, and practiced repeating prompts delivered through headphones. The experiment itself took place in a small PVC-and-felt tent intended to minimize light, noise, and distraction (*SI Appendix*, Fig. S2). Paired visual stimuli were presented on two 35.03 × 20.93 cm 720p LED displays (AOC) arranged approximately 10 cm apart, and angled so that both were maximally visible to the infant. Infants sat on their mothers’ laps inside the tent, centered before the table upon which the two displays were placed at roughly infant eye level. Caregivers received the auditory prompts over Bluetooth-connected headphones (Bose QCII), while a webcamera (Razer Kiyo) centered between the displays recorded infants’ gaze at 720p, 60 FPS, for later manual coding. The experimenters sat behind a felt curtain that served as a backdrop for the displays. Following two familiarization trials (fireworks on one screen, then on the other), the critical trials began. Optional attention-getting trials (static images of fireworks on both displays) were presented every four trials, and supplemented by one of the experimenters poking a multicolored rattle out from the bottom edge of the felt curtain and shaking it until the infant looked to the screens again.

On each trial, a bell announced the appearance of the paired visual stimuli. Caregivers—whose eyes were closed throughout the study—heard the sentence prompt for the trial over their headphones, followed by a second bell audible only to them, which signaled their turn to repeat the prompt (Fig. 5). The experimenter controlled the presentation of the stimuli from the other side of the curtain, and relied on a live feed to time the onset of each trial for when the infant was calm and attentive.

#### Manual gaze coding and analysis.

Infants’ left/right gaze durations on each trial were coded in Datavyu (104) by trained research assistants (see *SI Appendix*, Text for more detail). An automated Ruby (3.0) script computed multiple descriptive metrics from the Datavyu files, including infants’ total left- and right-looking time durations during the prenaming period (0 to 367 ms post target noun onset), and analysis window (367 to 3,500 ms postonset). We excluded at the trial-level if the infant never looked to the displays during the prenaming period, was noted as actively crying by coders, looked for less than one-third of the analysis window (3,133/3 ms), or if the caregiver or experimenter erred in some way (87). Trials often met multiple exclusion criteria simultaneously. In Experiment 1, a total of 93 trials from 20 infants were excluded (72 where the infant looked for less than one-third of the analysis window). Following previous work, we excluded at the subject-level if infants failed to contribute useable data on at least half of all test trials (n=0 in Experiment 1).

### Experiment 2.

Experiment 2 used the same procedures as Experiment 1, but tested knowledge of Tseltal honorific greeting terms (Fig. 5).

#### Participants.

Twenty-one mother–infant dyads speaking and learning Tseltal as their primary language participated in Experiment 2 (N=21 infants, 5.33–14.93 mo, *M*_age_ = 10.77 mo, SD_age_ = 2.67; N=21 mothers, 14–42 y, *M*_age_ = 26.81 y, SDage = 7.80 y), 17 of whom had previously participated in Experiment 1 (5.33–14.93 mo, *M*_age_ = 10.85 mo, SD_age_ = 2.64 mo). The infants not shared with the sample from Experiment 1 had also not yet begun walking, and their mothers reported rarely removing them from their slings throughout the day. We excluded 47 trials (25 for $<\frac{1}{3}$ looking during the analysis window) from 9 infants for meeting one or more of the above exclusion criteria; as a result, 3 infants’ data were excluded from analysis, based on contributing useable data on less than half of test trials.

#### Stimuli.

Visual and auditory stimuli were designed to test infants’ knowledge of the four core honorifics: *me’tik*, *tatik*, *kantsil*, *tatil*. Honorifics were paired to create two honorific-pairs whose members contrasted in sex and in age: OLD WOMAN–YOUNG MAN and OLD MAN–YOUNG WOMAN. Potential target images were identified via online search, then culled and validated by a sample of Tseltal adults (N=9) to confirm that each image uniformly elicited the intended term. Two photos for each honorific were then edited to produce standardized visual stimuli with the target individual isolated and centered on a monochrome gray background (eight total images; Fig. 2 and *SI Appendix*, Text). We formed four image-pairs (two for each honorific-pair), within which the size, angle, crop, and brightness of the portraits were roughly equated.

Auditory stimuli were recorded by a female adult speaker of Tseltal, who was instructed to produce each honorific as if greeting someone on the road. The resulting prompts were standardized for volume and length (1 s). A total of eight test trials, combining prerecorded prompts and image-pairs, were presented in a pseudorandom order. These trials appeared in the first block of a longer study of infants’ knowledge of the greeting routine, the other trial types for which we do not report on here.

## Supplementary Material

Appendix 01 (PDF)

## Data Availability

Anonymized data (infant looking time data and study stimuli) have been deposited in SI Appendix for Infants who are rarely spoken to nevertheless understand many words (osf.io/kcza9) (105). Analysis code can be found at https://github.com/foushee/tseltal-infants (106).

## Footnotes

^*^We use “child-directed language” to refer to any language that is directed to the target child—whether by an adult or another child. We use “other-directed language” to refer to any language—from an adult or another child—that is within earshot of the target child, but is intended for another listener.

^†^Many prior studies have also shown that young children can learn the forms and/or partial meanings of new words via passive exposure to disembodied speech (56–58). Although these studies are suggestive of what children may be able to learn via overhearing, how they translate to real-world contexts remains unclear. For example, the stimuli presented to infants in these studies necessarily provided concentrated evidence for a particular linguistic generalization (e.g., by repeatedly presenting the same set of novel words simultaneous with their potential referents), while real-world other-directed language is likely to provide evidence that is substantially “noisier” and less tailored to the learner (41, 59). Moreover, infants in these studies typically had little else to attend to besides the speech and stimuli presented to them and—in the absence of other potential addressees—may even have assumed that the speech was being directed to them.

^‡^Also relevant to understanding the nature of the language directed to Tseltal infants is the possibility that a large portion of this input may derive from other children as opposed to adults. For example, one study found that 64% of input directed to Yucatec Mayan 13-mo-olds was from other children (65). Compared to language from adults, childdirected language from children tends to be less structurally complex (26, 66) and lexically diverse (67), less contingent on and responsive to the target child’s vocalizations (68), less adapted to the target child’s stage of language development (68), and more focused on controlling the target’s child behavior than engaging them in conversation (26, 69, 70). These dimensions of variability have been linked to growth in linguistic competence across development, including in infancy (e.g., responsiveness has been linked to the size of infants’ vocabularies and the timing of language milestones; 16). Thus, to the degree that infants in our sample received a high proportion of their child-directed language from other children, this could provide further support for the claim that Tseltal infants receive little of the “high-quality” input that is believed to drive language learning. Still, future work should address the role of child-directed language from other children and how it might vary across different sociocultural contexts (65, 71).

^§^Current computational models which formalize the learning of a word as a function of accumulating experience with that word in child-directed language also predict that learning trajectories should be significantly delayed when a child has received less directed speech (60, 91).

^¶^All Wilcoxon tests are two-sided with μ=0; all binomial tests are two-sided with P=0.5.

^#^However, after excluding data from our oldest participant—who showed notably low evidence of word recognition (*mean difference score* =0.02)—subject-level scores exhibited a significant (α=0.05) positive correlation with infants’ age in months (τ=0.33, P=0.04).

^‖^This analysis enabled us to include n=94 additional trials that had to be dropped from the paired-difference score analyses, representing 18% of all nonexcluded trials.

^**^Notably, this method is arguably even more ecologically valid for Tseltal infants than the method of Experiment 1, because mothers’ speech is directed to the addressees of the greetings, rather than to the children themselves.

^††^Three infants lacked sufficient yoked trial data to allow us to compute a difference score for either honorific-pair; see *SI Appendix*, Text for details.

^‡‡^We note that even in this scenario, children in child-centered contexts may derive some linguistic knowledge from overhearing. For example, there are some aspects of language use—referential pronouns like “you” and “I” (95), curse words, prosody, and complicated syntactic structures—which may be easiest to learn for children of *all* linguistic communities by observing conversations between more adept language users.

^§§^Because other-directed speech has typically been defined in the negative as any and all speech which is not child-directed, it constitutes a heterogeneous category. For example, other-directed speech could include speech directed to an adult, or to a different child; it could be spoken by a caregiver, a sibling, or a stranger; it could be more or less linguistically complex than the speech typically directed to the child; and it could vary in its auditory quality and the availability of cues to the speaker’s referential intent. Such heterogeneity could help explain why previous studies have failed to detect correlations between the quantity of other-directed speech in children’s environments and their subsequent vocabulary growth (65, 88).
