# Supplementary material for: Infants who are rarely spoken to nevertheless understand many words
Source: Proc Natl Acad Sci U S A. 2024 May 30;121(23):e2311425121. doi: 10.1073/pnas.2311425121 (PMC11161804; doi:10.1073/pnas.2311425121)
Supplement: Supplementary file 1 — Appendix 01 (PDF) [file pnas.2311425121.sapp.pdf]

Supporting Information for

Infants who are rarely spoken to nevertheless understand many words

Ruthe Foushee, Mahesh Srinivasan

Ruthe Foushee
E-mail: ruthe@newschool.edu

This PDF file includes:

Supporting text
Figs. S1 to S5
Tables S1 to S6
SI References

Supporting Information Text

More Information on Honorific Greetings. Greetings within this community follow a call-and-response format wherein adults exchange honorific terms based on sex, age, status, and sometimes kinship. Honorific greetings occur whenever community members encounter one another, including when passing in the road, when joining a shared car, or when calling from the edge of a property to announce their presence to its inhabitants. The core honorific terms are each two syllables long: *me'tik* (for an older woman), *tatik* (for an older man), *kantsil* (for a younger woman), and *tatil* (for a younger man), and are delivered with a dipping intonation with stress and variable degrees of lengthening on the second syllable (for sample recordings, see 1). Adults greet each person in a group individually; each recipient responds with the appropriate honorific using the same intonation. In greeting, honorific terms are also often produced at a high volume, as they are frequently used between community members greeting each other from a distance outdoors. As these honorifics are the basic address forms, they are also used without the drawn-out intonation whenever politely addressing a Tseltal person, and can be used to politely reference people. Tseltal honorifics are thus likely to be highly salient linguistic items, even for a learner with limited visual access and limited language knowledge. Critically, infants are excluded from the greeting routine: there is no honorific for addressing a baby. Thus, any recognition of these honorific greetings necessarily reflects language knowledge learned through overhearing.

Method

Participant Recruitment. To recruit eligible participants, a research assistant from the community and the first author called at family compounds within a two-hour hike of the testing location. Mothers with young infants were often summoned by family members or neighbors, and if they expressed interest in the study, were typically scheduled to participate the following day. When asked at this initial contact, mothers often provided an approximate age for their infants; they were encouraged to look for evidence of infants' exact birth dates—or as near an estimate as possible—before their scheduled participation. In cases where the child's birth date remained uncertain, as when the child was known to have been officially registered several weeks or months after their actual birth, a specific date of birth was estimated by triangulating the child's birth relative to those of infants born during the same period (mothers of similar-aged infants often remembered whether another infant from the same community was born before or after their own child). We sought to enroll approximately 30 infants, to match the sample size in Bergelson & Swingley (2012). We successfully recruited 33 infants, but only tested infants on the procedures reported in the main text if their caregivers reported that infants almost exclusively heard Tseltal at home, and were not yet walking (the latter criterion a reflection of prior ethnographic work linking the onset of walking to increases in child-directed language).

The consent procedure we used was the product of previously-conducted informational interviews with mothers in the community, and provided a structured format for participants to specify their comfort level with different potential forms of data (e.g., text transcripts, voice recordings, photos and videos including their or their children's faces) and different potential audiences (e.g., community members, local versus foreign academics and students, etc.). Given that mothers' primary associations with having to sign a physical document (typically just with an "X") were the doctor's office and bank—both contexts with amplified colonial power dynamics—consent for each task was given verbally (elicited in Tseltal by a local research assistant).

Paired-Picture Stimuli Preparation. All visual and auditory stimuli can be found at the online repository for this study (1, 2).

Experiment 1. Nouns in Experiment 1 were drawn from responses on initial interviews with mothers ($N = 16$) regarding Tenejapan infancy, including the first solid foods that infants eat, the beverages that they first sip, the animals that they are most frequently around, and the first recognizable words (and conventionalized animal sounds) that they eventually produce. Responses were highly consistent across caregivers (who themselves were often reporting on observations and experiences across multiple children). We next collected multiple images for the nouns that had been repeatedly mentioned across caregivers, and, with feedback from a local research assistant, narrowed the images down to 2–4 per noun. A sample of adults ($N = 9$) was then asked to name these images, and we used only those nouns and images that elicited the target noun as the first or second word that adults produced. Next, we excluded nouns whose images translated poorly to the digital displays, often because naturalistic examples of them were difficult to uniquely identify, had a difficult scale to convey without context, or were too dark, monochromatic, or low-contrast (e.g., *chenek*—"beans").

Following (3), we constructed candidate noun-pairs by pairing one animate (or animate-adjacent, in the case of *tep*—"shoe") and one inanimate noun, avoiding pairing nouns with matching onsets (e.g., *wakax* and *waj*), and trying where possible to match syllable counts and approximated levels of salience and/or frequency, informed by mothers' reports. For example, we paired the exciting and apparently early-produced *ts'i'*—"dog" with *k'ajk'*—"fire," rather than with *ja'*—"water" (a notably less thrilling referent). We used our set of named images to construct image-pairs that were visually equated in terms of area and brightness, which we then presented to infants ($N = 2$) without audio to ensure that infants looked at *both* images when they were presented side-by-side. Infants almost never looked at the images for *tuts'*—"spoon" (then paired with *wakax*—"cow"), and overwhelmingly looked at the images for *karo*—"car" (then paired with *mayil*—"squash"), leading us to pair *wakax* and *ch'uhmte'e* ("cow-squash" [chayote]), and to introduce *tep*—"shoe" as the distractor for *karo*. Looking-time preferences for this final set were measured with one infant before being finalized.

We recorded audio prompts for all four carrier phrases with each noun, before constructing a single ordered list of trials such that: (a) each noun appeared with two distinct carrier phrases, (b) each carrier phrase was used an equal number of times ($n = 8$ trials), and (c) adjacent trials neither used the same carrier phrase nor tested the same noun-pair.

Experiment 2. Basic honorific terms were identified via observation and consultation with local research assistants. We collected a set of potential target faces via online image searches, and culled it in collaboration with research assistants, before asking same sample of adults cited above ($N = 9$) how they would greet each pictured addressee. We use only images that uniformly elicited a single honorific greeting (i.e., no matter the age or status of the 'greeter'). Sample audiorecordings of adults producing honorifics as greetings for our face stimuli can be found at the online repository for this study (1). In both previous informational interviews and the stimuli validation task, we also asked Tseltal adults how they would greet an infant. This question was nearly uniformly met with laughter.

To produce the critical trials, the same female voice recorded all four honorific greeting prompts, which were paired with the image-pairs and presented in a single pseudorandom order across children, mindful to avoid immediate repetitions of the same image-pairs or target honorifics.

Manual Gaze Coding. To prepare for the manual coding of infants' gaze, we first clipped the raw study-session videos into individual test trials, starting and ending with the appearance of the visual stimuli on the displays. Trained research assistants used Datavyu (4) to code these pre-clipped trial videos in three serial 'passes' (5), conducted over the entire set of clips. In the first coding pass, research assistants segmented the video clips into two or three consecutive periods, depending on experiment: (1) from the appearance of the visual stimuli to the onset of the mother's speech ("pre"), (2; only for Experiment 1) from the onset of the mother's speech to the onset of the target noun ("sp"), and (3) from the onset of the target word to the end of the trial ("target"). At the end of the first pass, research assistants hid the columns they had just coded. The second and third coding passes were completed without sound. In them, research assistants coded infants' leftward looks over the entire duration of the trial (second pass), then infants' rightward looks over the entire duration of the trial (third pass). See online repository for video-coding manual.

Pair-based Mean Difference Score Calculation. To clarify the calculation of the pair-based mean difference score, we will use Fig. S1, which illustrates the trials testing the noun-pair *alal-ixim*—"baby–corn." Infants saw two *alal-ixim* image-pairs: in one, BABY-A appears on the left, with CORN-A on the right. In the other, CORN-B appears on the left, with BABY-B on the right. Each image-pair is presented twice, both times using the same carrier sentence: in one presentation, the target word will be *alal* ("baby"), and in the other, the target word will be *ixim* ("corn"). The four trials corresponding to each noun pair are separated by interleaved trials testing other noun-pairs (the actual position of the four *alal-ixim* trials in the complete list of trials that infants saw is noted via the TRIAL # labels in Fig. S1).

Importantly, the difference in fixation proportions is always computed within image-pair, meaning, based on trial-level data using identical visual stimuli and the same carrier sentence. Thus, with useable data for all four trials testing *alal-ixim*, an infant's pair-based difference score for the noun-pair would be the mean of two values: (1) the proportion of the infant's gaze during the analysis window, which was directed to BABY-A when the prompt was *banti ay te* ***alal****?*—"where is the **baby**?" minus the proportion of the infant's gaze during the analysis window, which was directed to BABY-A when the prompt was instead *banti ay te* ***ixim****?*—"where is the **corn**?" and (2) the proportion of the infant's gaze during the analysis window, which was directed to BABY-B when the prompt was *ilawil te* ***alal***—"look at the baby," minus the proportion of their gaze during the analysis window, which was directed to BABY-B when the prompt was instead *ilawil te* ***ixim***—"look at the corn."

Given the pair-based nature of this calculation, as soon as an infant is missing (or has had excluded) data for one of the four trials, the data for the *other* trial presenting the same image-pair and carrier sentence will be dropped as well. In this situation, the infant's overall difference score for the noun-pair will reflect the difference score computed for just one image-pair. While a pair-based (unaveraged) difference score remains calculable for a given noun-pair when only a single trial is missing, it can be impossible to calculate as soon as infants are missing two trials, and the two trials that remain are from across image-pairs (that is, if one trial is from the BABYA-CORNA image-pair, and the other is from the BABYB-CORNB image-pair). Both remaining trials will also be dropped. This level of detail is intended to illustrate the value of our second analysis, which—by seeking evidence of word recognition in infants' *within-trial* looking time preferences—enables us take advantage of *all* non-excluded trials, regardless of whether useable data also exists for their 'matches.'

Results

Data Inclusion.

Experiment 1. In Experiment 1, there were 16 data points/infant for the first, pair-based analysis (1 paired difference score for each image-pair, 2 image-pairs/noun-pair; *observed range*$= 2 - 16$, $M = 10.33$, *Med*$= 10$, *Mode*$= 10$), and up to 32 data points/child for the second, trial-phase logistic regression analysis (1 data point for each trial; *observed range*$= 16 - 32$, $M = 25$, *Med*$= 26$, *Modes*$= 16, 25, 31$). That is, we were able to use 94 trials in the second analysis that we were forced to drop in the first (out of 528 trials total; 18% of all trials).

Experiment 2. In Experiment 2, there were up to 4 data points/child for the pair-based analysis (1 paired difference score for each image-pair, 2 image-pairs/honorific-pair; *observed range*$= 1 - 4$, $M = 2.88$, *Med*$= 3$, *Modes*$= 2, 4$), and up to 8 data points/child for the second, trial-phase logistic regression analysis analysis (1 data point for each critical trial; *observed range*$= 3 - 8$, $M = 6$, *Med*$= 6$, *Mode*$= 8$). We were thus able to 'redeem' data from 25 trials (out of 115 trials total; 22% of all trials), and three children (who had useable data on a combination of trials that precluded computing a mean difference score for either honorific-pair).

Trial Phase and Looking Time Durations.

Experiment 1. Trials in Experiment 1 were on average 14.86s (*95% CI*= [14.60, 15.13]) long (*range*= 5.42 − 24.82s, $M = 14.86s$, *Med*= 14.65s), and infants spent an average of 6.70s [6.55, 6.84] total looking at the displays (*range*= 1.97 − 12.17s, *Med*= 6.77s), or between 0.07 and 0.99 of the total trial duration; $M = 0.46$ [0.45, 0.48]). The pre-naming window was between 3.12s and 9.44s ($M = 5.22s$, *Med*= 4.97s). Infants tended to spend a greater proportion of the post-naming period looking at the displays (PRE-NAMING *range*= 0.03 − 1.00, $M = 0.77$, *Med*= 0.84; POST-NAMING *range*= 0.34 − 1.00, $M = 0.87$, *Med*= 0.94).

Experiment 2. Trials in Experiment 2 were 14.29s (*95% CI*= [13.75, 14.82]) long on average (*range*= 4.34 − 20.99s, *Med*= 14.49s), and infants spent an average of 11.44s (*95% CI*= [10.81, 12.10]) total looking at the displays (*range*= 3.29 − 31.13s, *Med*= 11.91s, or between 0.32 and 1.00 of the total trial duration ($M = 0.79$, *95% CI*= [0.76, 0.82]; *Med*= 0.83). The pre-naming window was between 2.31s and 6.12s long ($M = 3.63s$, *Med*= 3.48s). Infants tended to look at the displays for a greater proportion of the post-naming phase, relative to the pre-naming phase (PRE-NAMING *range*= 0.12 − 1.00, $M = 0.74$, *Med*= 0.78; POST-NAMING *range*= 0.38 − 1.00, $M = 0.90$, *Med*= 0.96; see Figure S4 for the distribution of looking times during the two trial phases).

Modelling Pair-Based Mean Difference Scores. As reported in brief in the main text, we modeled infants' difference scores to ensure that our positive impression of infants' performance was not driven by a few particularly high-scoring infants. Specifically, we fit linear mixed effects models to children's difference scores for all item-pairs, including random intercepts for each subject and item to account for individual- and item-level variability. Null models were fit using the `lmer` package in R (6, 7), using the following syntax: `lmer(difference_score ∼ 1 + (1|subject) + (1|item_pair))`. In both experiments, models with this random effects structure resulted in a singular fit. Thus, we refit the models without the random intercepts for item (which showed zero variance): `lmer(difference_score ∼ 1 + (1|subject))`. We used the `anova` function to compare these models to models without an intercept (`lmer(difference_score ∼ 0 + (1|subject))`. Code for all analyses and plots (8)—here and in the main text—can be found at https://github.com/foushee/tseltal-infants (2).

Experiment 1. As reported in the main text, we can interpret the intercept of the mixed effects model as the expected value of children's scores. The intercept was significantly positive ($\beta_0 = 0.10$, *95% CI:* [0.03, 0.17], $t(21.45) = 2.77$, $p = 0.011$, *Cohen* $d = 0.28$; see Table S3), and of similar magnitude to our unadjusted calculation of the mean, suggesting that our results are not driven by the robust word recognition of a few exceptional participants.

Experiment 2. The intercept for the model fit to the data for Experiment 2 also suggests that infants' performance was on-average positive, taking into account subject-level variability in performance ($\beta_0 = 0.14$, *95% CI:*[−0.01, 0.29], $t(15.23) = 1.98$, $p = .066$, $\chi^2(1) = 3.53$, $p = 0.060$, *Cohen* $d = 0.53$; see Table S4), though this did not reach significance (at the $\alpha = 0.05$ level).

Modeling the Ratio of Target/Non-target Looking in Pre-naming and Post-naming Windows. We follow (3) in complementing our pair-based measure of word recognition with an analysis that enabled us to analyze *all* of the (non-excluded) trial-level data for each infant. Specifically, we fit mixed effects logit models to infants' ratios of target versus non-target looking times (in number of 20ms bins) on each trial, with trial phase (PRE-NAMING/POST-NAMING, *reference level*=PRE-NAMING) as a fixed effect, and random intercepts for subject and item. The PRE-NAMING phase was defined as from the onset of the visual stimuli to 366 ms after the onset of the target word, and the POST-NAMING phase was defined as from 367 to 3500 ms after the onset of the target word. We fit these models using the `lme4` package in R (6, 7) using the following syntax: `glmer(cbind(num_target_20ms_bins, num_nontarget_20ms_bins) ∼ 1 + trial_phase + (1|subject) + (1|item_pair), family="binomial")` (Model 1 in Tables S5 and S6). In the case of a singular fit, as when fitting the model to the data for Experiment 2, we drop the random intercept for item, and refit the model.

Below and in the main text, we exponentiate the coefficients of these models to report the effects in terms of odds ratios (raw—unexponentiated—coefficients can be found in Tables S5 and S6). We additionally report odds ratios for models fit to the data for all infants and updated to explore the effect of age (in months, mean-centered) on children's target-/non-target looking times: `glmer(cbind(num_target_20ms_bins, num_nontarget_20ms_bins) ∼ 1 + trial_phase + age_months + trial_phase:age_months + (1|subject) + (1|item_pair), family="binomial")` (Model 3 in Tables S5 and S6). Odds ratios for trial phase that are greater than 1 (positive model coefficients) are consistent with word knowledge, as they indicate that infants showed a greater looking time preference for the target image after hearing the target word than they had before hearing it. Similarly, odds ratios greater than one for the interaction between trial phase and child age (POST-NAMING:Age) suggest increasing word recognition with age, such that older infants showed a greater positive effect of trial phase than did younger infants. Finally, we obtain significance levels for each parameter via Wald tests (using the `Anova` function in the `car` package; 9).

Experiment 1. Of the three models we tested (Table S5), the best-fit model showed a reliable effect of trial phase (POST-NAMING *OR*= 1.29, *95% CI:* [1.26, 1.31], *Wald* $\chi^2(1) = 635.84$, $p < .001$, *Cohen* $d = 0.76$) and interaction with age, suggesting that older children showed an even greater increase than younger children in their ratio of target to non-target looking after hearing the target noun (*OR*= 1.08, *95% CI:* [1.07, 1.09], *Wald* $\chi^2(1) = 401.02$, $p < .001$, *Cohen* $d = 0.23$; see Table S5).

.

Experiment 2. The model with the full random effects structure resulted in a singular fit, so we refit a model without the random intercepts for honorific-pair. As in Experiment 1, the best-fit model of the three we tested (Table S6) included trial phase, infant age (in months, centered), and the interaction between trial phase and infant age. Here again the effect of trial phase was significant ($OR = 1.31$, *95% CI:* [1.25, 1.38]; *Wald* $\chi^2(1) = 123.84$, $p < .001$, *Cohen* $d = 0.65$), as was the effect of age, suggesting that older infants tended to prefer the target less than younger infants before hearing the honorific greeting term ($OR = 0.90$, *95% CI:* [0.84, 0.97]; *Wald* $\chi^2(1) = 4.49$, $p < .05$; $d = 0.25$). The (weak) interaction with age that we observe suggests that the older children in our study showed a somewhat greater increase in the ratio of target:non-target looking after hearing the target honorific ($OR = 1.03$, *95% CI:* [1.02, 1.05], *Wald* $\chi^2(1) = 13.82$, $p < .001$, *Cohen* $d = 0.08$).

References

1. R Foushee, Online supplementary materials for infants who are rarely spoken to nevertheless understand many words (2023) osf.io/kcza9. Deposited 10 August 2023.
2. R Foushee, tseltal-infants (2024) https://github.com/foushee/tseltal-infants. Deposited 10 August 2023.
3. E Bergelson, D Swingley, At 6–9 months, human infants know the meanings of many common nouns. *Proc. Natl. Acad. Sci.* **109**, 3253–3258 (2012).
4. Databrary Project, *Datavyu: A Video Coding Tool. Databrary Project, New York University* (New York University), (2014).
5. Databrary Project, *Best Practices for Coding Behavioral Data from Video.* pp. 99–112 (2014) Available at https://datavyu.org/docs/user-guide.pdf (accessed 14 March 2020).
6. R Development Core Team, *A Language and Environment for Statistical Computing.* (R Foundation for Statistical Computing, Vienna, Austria) Vol. 2, pp. https://www.R–project.org (2020).
7. DJ Barr, R Levy, C Scheepers, HJ Tily, Random effects structure for confirmatory hypothesis testing: Keep it maximal. *J. Mem. Lang.* **68**, 255–278 (2013).
8. H Wickham, *ggplot2: Elegant Graphics for Data Analysis.* (Springer-Verlag New York), (2016).
9. J Fox, S Weisberg, *An R Companion to Applied Regression.* (Sage, Thousand Oaks CA), Third edition, (2019).

Table S1. Experiment 1 Item Means

noun-pair	*n*	*M*	95% CI	min	max
baby-corn	21	0.03	(-0.16, 0.22)	-0.79	1.00
shoe-car	19	0.15	(-0.08, 0.36)	-1.00	1.00
cow-squash	19	0.11	(-0.12, 0.34)	-1.00	1.00
chicken-tortilla	21	0.04	(-0.21, 0.30)	-0.90	1.00
dog-fire	21	0.22	(0.04, 0.40)	-0.45	1.00
horse-soda	21	0.23	(0.03, 0.42)	-0.56	0.77
rabbit-soup	21	0.13	(-0.08, 0.32)	-0.65	0.96
sheep-water	21	0.05	(-0.11, 0.23)	-0.61	0.72

Table S2. Experiment 2 Item Means

honorific-pair	n	M	95% CI	min	max
old man-young woman	13	0.08	$(-0.14, 0.29)$	-0.71	0.54
old woman-young man	15	0.18	$(0.07, 0.30)$	-0.19	0.54

Table S3. Linear Mixed Effects Model of Paired Difference Scores in Experiment 1

	Model 1
(Intercept)	0.12^{**}
	(0.04)
AIC	176.16
BIC	184.99
Log Likelihood	-85.08
Num. observations	140
Num. groups: subject	21
Var: subject (Intercept)	0.00
Var: Residual	0.19

$^{***}p < 0.001$; $^{**}p < 0.01$; $^{*}p < 0.05$

Table S4. Linear Mixed Effects Model of Paired Difference Scores in Experiment 2

	Model 1
(Intercept)	0.14^{*}
	(0.07)
AIC	20.60
BIC	24.60
Log Likelihood	-7.30
Num. observations	28
Num. groups: subject	15
Var: subject (Intercept)	0.04
Var: Residual	0.07

$^{***}p < 0.001$; $^{**}p < 0.01$; $^{*}p < 0.05$

Table S5. Mixed Effects Logit Models of Target vs. Non-target Looking in Experiment 1

	Model 1	Model 2	Model 3
(Intercept)	0.03	0.03	0.03
	(0.07)	(0.07)	(0.07)
Phase:POST-NAMING	0.25^{***}	0.25^{***}	0.25^{***}
	(0.01)	(0.01)	(0.01)
Infant Age (months, centered)		0.00	−0.03
		(0.02)	(0.01)
POST-NAMING:Age			0.08^{***}
			(0.00)
AIC	114953.46	114955.35	114555.51
BIC	114973.31	114980.17	114585.28
Log Likelihood	−57472.73	−57472.68	−57271.75
Num. observations	1056	1056	1056
Num. groups: subject	21	21	21
Num. groups: noun-pair	8	8	8
Var: subject (Intercept)	0.03	0.03	0.03
Var: noun-pair (Intercept)	0.02	0.02	0.02

$^{***}p < 0.001$; $^{**}p < 0.01$; $^{*}p < 0.05$

Table S6. Mixed Effects Logit Models of Target vs. Non-target Looking in Experiment 2

	Model 1	Model 2	Model 3
(Intercept)	0.12	0.09	0.09
	(0.11)	(0.10)	(0.10)
Phase:POST-NAMING	0.26^{***}	0.26^{***}	0.27^{***}
	(0.02)	(0.02)	(0.02)
Infant age (*months*, centered)		-0.09^{*}	-0.10^{**}
		(0.04)	(0.04)
POST-NAMING:Age			0.03^{***}
			(0.01)
AIC	19928.81	19926.32	19914.50
BIC	19939.13	19940.07	19931.69
Log Likelihood	-9961.41	-9959.16	-9952.25
Num. observations	230	230	230
Num. groups: subject	18	18	18
Var: subject (Intercept)	0.22	0.17	0.17

$^{***}p < 0.001$; $^{**}p < 0.01$; $^{*}p < 0.05$

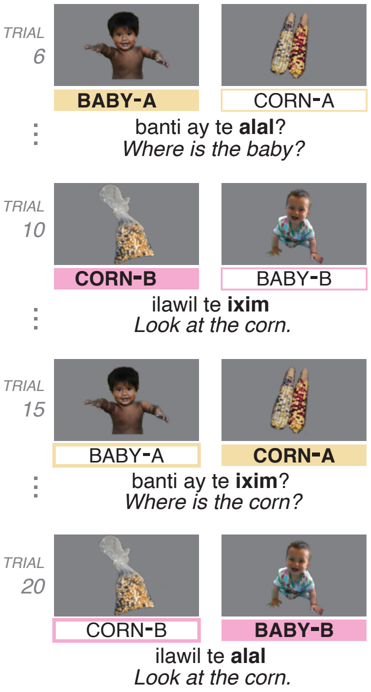

Fig. S1. *Visual Schematic of Trials Corresponding to Single Noun-Pair.* Trial numbers shown on the left reflect the true sequential position of the trials as they were presented to infants, interleaved with trials testing other noun-pairs. Difference scores for a given infant are computed first within each image-pair, then averaged to obtain a single measure of word recognition at the level of the noun-pair. Thus, a difference score for the noun-pair *alal–ixim* ("baby–corn") can be computed only if an infant provides useable gaze data on TRIAL 6 and TRIAL 15, and/or on TRIAL 10 and TRIAL 20 (but not if the infant only provides useable gaze data on, e.g., TRIAL 6 and TRIAL 10, or TRIAL 6 and TRIAL 20).

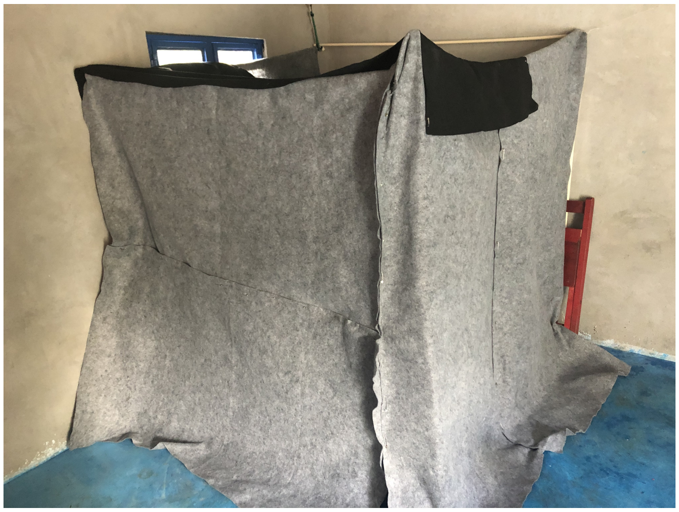

Fig. S2. *Field Experiment Booth.* The experiment took place inside a booth constructed with PVC pipe and industrial felt, intended to control the amount of light and dampen outside noise. Experimenters sat on one side of an interior felt curtain; mothers and their infants sat on the other.

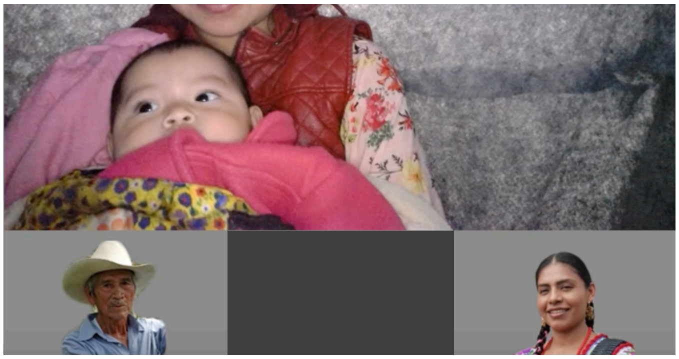

Fig. S3. *Example Honorific Paired-Picture Trial from Experiment 2.* Video still taken from a trial testing honorific-pair *tatik–kantsil* (OLD MAN–YOUNG WOMAN). Here, the infant is looking to the appropriate addressee—the older man—after hearing her mother deliver the honorific greeting *tatik*.

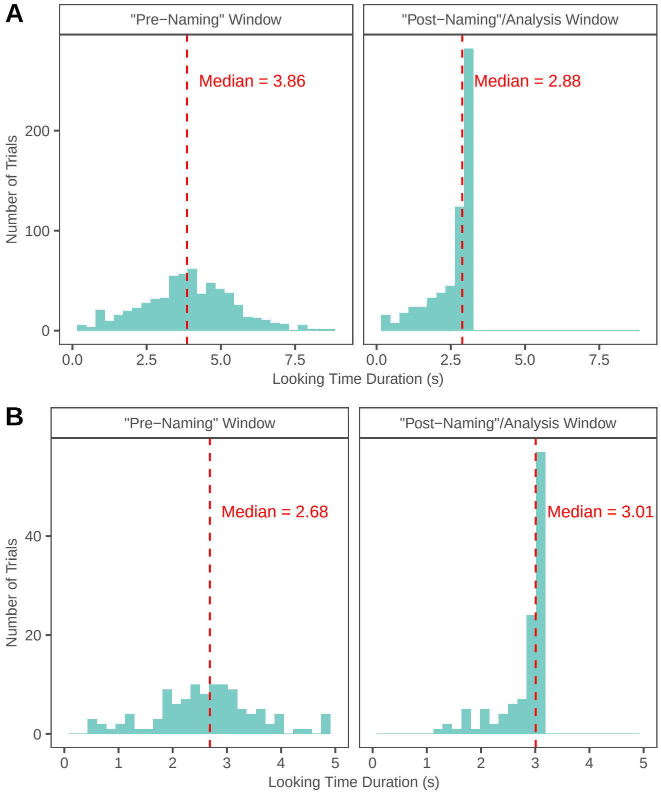

Fig. S4. Looking Time Durations (in s) by Trial Phase in Experiments 1 (top) and 2 (bottom). Vertical dashed lines mark the median duration, labeled in s The analysis window (second panel in each row) was $3133ms$ long; trials where infants looked for less than one-third of its duration ($1044ms$, or $1.04s$) were excluded.

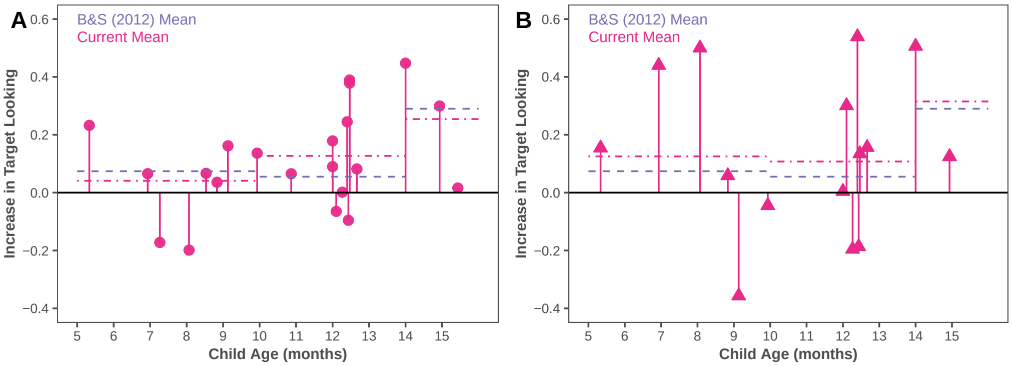

Fig. S5. *Mean Difference Scores by Subject in Experiments 1 (left) and 2 (right). Average scores for three age groups of infants in the current studies are plotted with horizontal magenta dot-dashed lines, for visual comparison with average scores from (3), plotted with horizontal purple dashed lines.*
